# Effectiveness of Adjunctive Measures to Limit Recurrence and Reoperation After Laparoscopic Repair of Large Paraesophageal Hernias: A Single-Institution Series

**DOI:** 10.3390/jcm15072741

**Published:** 2026-04-04

**Authors:** Julia Kessel, Dimitrios N. Varvoglis, Timothy Feeney, Madeleine Higgins, Justin Hsu, Lauren M. Cook, Chris B. Agala, Maggie M. Hodges, Timothy M. Farrell

**Affiliations:** 1 School of Medicine, University of North Carolina at Chapel Hill, Chapel Hill, NC 27599, USA; 2 Department of Orthopaedic Surgery, University of Toledo, Toledo, OH 43606, USA; 3 Department of Surgery, University of North Carolina at Chapel Hill, Chapel Hill, NC 27599, USA; 4 Department of Surgery, University of Colorado Anschutz Medical Campus, Aurora, CO 80045, USA; 5 Department of Epidemiology, University of North Carolina at Chapel Hill, Chapel Hill, NC 27599, USA; 6 Department of Surgery, Morton Hospital-Brown University Health, Taunton, MA 02780, USA; 7 Department of Surgery, University of Toledo, Toledo, OH 43606, USA

**Keywords:** laparoscopy, giant hiatal hernia, paraesophageal hernia repair, PEG, gastrostomy, gastropexy, mesh, fundoplication

## Abstract

**Background:** Despite surgical repair, large paraesophageal hernias (PEHs) often recur. To minimize recurrence, adjunctive measures, such as mesh and gastropexy, have been explored, but their impact on recurrence and reoperation rates remains unclear. Therefore, we analyzed our single-institution case series, where absorbable hiatal overlay mesh and percutaneous endoscopic gastrostomy (PEG) placement were utilized systematically. **Methods**: Patients undergoing laparoscopic large PEH repair by a single surgeon between 1 January 2006 and 31 May 2021 were identified. Demographic data, hernia size, number of hiatal sutures used, use of mesh and/or PEG, fundoplication type, and complications were extracted by retrospective chart review. Hernia recurrence was assessed though postoperative radiographic and endoscopic studies or need for reoperation. Fisher’s exact, chi-square, Mood’s two-median and t-tests were used for between-group comparisons. Generalized linear models were used to assess associations between mesh and PEG placement and number of hiatal sutures and to compare risk differences for recurrence between treatment types (partial versus complete fundoplication; mesh versus no mesh; and PEG versus no PEG). Kaplan–Meier estimator with log-rank test was used to assess time to recurrence. **Results:** Overall, 413 patients (median age 66 years) underwent laparoscopic large PEH repair and fundoplication (51% partial and 49% complete fundoplications). Of these, 78% had overlay absorbable mesh and 51% had a PEG. With an average follow-up time of over 5 years, we found 19.9% had radiographic or endoscopic recurrence. Although cohort stratification based on mesh implementation and fundoplication type did not identify differences in recurrence rates, significantly lower recurrence rates were noted in patients with PEG compared to no-PEG (14.8% vs. 23.5%, *p* = 0.01). Notably, of five reoperations, all were in complete fundoplication patients, and one occurred in a patient with PEG placement at the index operation. **Conclusions:** PEG placement during laparoscopic PEH repair may lead to fewer recurrences in high-risk patients. Future prospective studies are warranted.

## 1. Introduction

Hiatal hernias (HHs) are a common condition, with large population-based studies indicating a prevalence of up to 17% among patients undergoing esophagogastroduodenoscopy (EGD) [1]. HHs are classified into four subtypes with sliding HH (type I) being most common [2]. HH types II-IV encompass the different paraesophageal hernias (PEHs). Anecdotally, it has been cited in the literature that PEHs constitute 5–10% of all HHs [3], however the true prevalence of the condition remains unknown. A recent analysis of the American College of Surgeons (ACS) National Surgical Quality Improvement Program database indicated that over the course of 8 years, almost 43,000 PEH repairs were included, with about 4% of them being emergent operations [4]. Most PEHs are repaired electively, because observation may lead to hernia incarceration and strangulation which requires a highly morbid emergent operation [4,5,6].

The surgical management of PEHs, which originally included a transthoracic approach, has evolved over the years [7]. The current standard treatment involves a transabdominal minimally invasive repair, which is preferred over transthoracic and open approaches because of reduced morbidity and length of hospital stay [8]. Unfortunately, recurrences may be seen in 10–59% of patients [9,10,11,12], with factors such as hernia and hiatus size potentially impacting the incidence [13].

To reduce recurrence, various techniques, such as use of mesh and gastropexy, have been proposed. The implementation of overlay absorbable mesh has been associated with fewer early radiographic recurrences [14,15], while gastropexy by gastrostomy placement may, according to the Society of American Gastrointestinal and Endoscopic Surgeons (SAGES) [8], “facilitate care in selected patients,” albeit being associated with an increased risk of superficial skin infections (SSIs) and pulmonary embolism (PE) [16].

Given the unclear impact of mesh and gastrostomy placement in minimizing PEH recurrence, we conducted a retrospective review of a prospectively maintained database to study the potential effect of fundoplication type, absorbable mesh and gastrostomy placement on PEH recurrence and reoperation rates in patients undergoing laparoscopic repair of large PEHs.

## 2. Methods

Cases of primary laparoscopic PEH repairs performed by a single surgeon from 1 January 2006 and 31 May 2021 at University of North Carolina (UNC) Hospitals were retrospectively reviewed. Patients were included if they underwent a repair of a large PEH, defined as a chronic herniation of more than 33% of the stomach, based on a prospectively maintained database and review of the operative note. Only type II-IV HHs were included. Patients were excluded if the case was done open, was a reoperation, or was an emergent presentation.

Data were extracted on: (1) patient demographics; (2) percentage of stomach herniated; (3) use of absorbable overlay mesh; (4) number of sutures used to close the hiatal defect; (5) percutaneous endoscopic gastrostomy (PEG) placement; (6) performance of secondary procedures; (7) complications (30-day overall and Clavien–Dindo [17] grade III-V); (8) reoperations and (9) recurrences. On follow-up, patients were defined as having a recurrence based on any report of a hiatal hernia on: (1) imaging performed more than 30 days postoperatively (typically a barium esophagogram); (2) postoperative upper gastrointestinal tract endoscopy; and/or (3) reoperation for hiatal hernia recurrence. For patients without follow-up imaging or endoscopy, no objective recurrence was assumed for the primary cohort analysis. Because postoperative radiographic or endoscopic evaluation was not performed routinely in all patients, we additionally performed a sensitivity analysis limited to patients who underwent postoperative radiographic and/or endoscopic follow-up. The follow-up time endpoint was determined based on the date of the last visit recorded as captured in the electronic medical record (EMR). Additionally, surveys capturing the postoperative quality of life were administered; however, responses were limited to most recent patients who did not overlap with those undergoing radiographic or endoscopic follow-up. For this reason, functional outcomes will be addressed in a separate, planned analysis.

### 2.1. Operative Technique

All patients with PEHs were managed electively, under general anesthesia, and using a 5-port laparoscopic approach. Key steps of the procedures included reduction in herniated contents, hernia sac dissection out of the mediastinum with excision of the hernia sac, circumferential mediastinal periesophageal dissection to achieve 2–3 cm intra-abdominal esophageal length, posterior (interrupted, pledgeted) crural closure using intra-corporeal suturing and knot tying. In certain cases where crural integrity was questioned, U-shaped biologic absorbable mesh (Biodesign Hiatal Hernia Graft—Cook Biotech Inc., West Lafayette, IN, USA) made from decellularized, multilayered porcine small intestine submucosa (SIS) was used as an overlay and secured with fibrin sealant (Tisseel—Baxter International Inc., Deerfield, IL, USA). In all cases, short gastric vessel mobilization and formation of a posterior partial or posterior complete fundoplication was performed. Usually, esophageal motility was not tested for large paraesophageal hernias given the difficulties in catheter placement and study interpretation, and these patients received a Toupet fundoplication by default. In cases of younger patients with more severe GER, a Nissen fundoplication was only fashioned if motility testing confirmed the percentage of esophageal peristalsis was >60% for wet swallows and distal esophageal amplitude was >30–40 mmHg or mean distal contractile integral was >600 mmHg-s-cm. When placed, a 20-French PEG was positioned using pull technique with laparoscopic visualization and was located on the greater curvature of the stomach at a point that would allow easy elevation of the anterior stomach to the left upper abdomen with limited traction after releasing the pneumoperitoneum at the end of the case. Patients were observed for 1–2 nights in hospital and were slowly advanced to a regular diet 4–6 weeks postoperatively. If a PEG was placed, patients were instructed in venting and daily flushing, and the tube was removed five to six weeks postoperatively, once diet and symptoms had returned to normal. PEG tubes were selectively used when one or more of the following were present: (1) >50% of the stomach was chronically intrathoracic; (2) the hiatal defect was large and/or closure was under appreciable tension; (3) crural muscle quality was judged intraoperatively to be poor or healing was considered at increased risk because of comorbidities or chronic immunosuppression; or (4) gastroparesis was a concern.

### 2.2. Hernia Risk Stratification

Percentage of intrathoracic stomach was used as the primary measurable parameter for assessment of hernia complexity and risk. In addition, the number of hiatal sutures required for crural closure was analyzed as a surrogate marker of defect size. Qualitative intraoperative assessments, including crural integrity and tension at closure, were also used to identify higher-risk hernias, although these remain surgeon-dependent measures that cannot be fully standardized retrospectively. It should be noted that 3 cm of intra-abdominal esophageal length was always achieved and therefore, Collis gastroplasty was not implemented in any of the patients in our cohort.

### 2.3. Statistical Analysis

Descriptive statistics were calculated as frequencies and percentages for categorical variables, and continuous variables were described by means and standard deviations (SD) or medians and interquartile ranges (IQRs). Between-group comparisons were completed using Fisher’s exact test, chi square test, Student’s *t*-test, and Mood’s two-median test. Additionally, generalized linear models were used to compare risk differences for recurrence between treatment types, including partial versus complete fundoplication, mesh versus no mesh, and PEG placement versus no PEG. Time to recurrence was the time from date of index paraesophageal hernia repair to diagnosis of a recurrence, defined as a recurrent hiatal hernia of at least 2 cm on either barium swallow or upper endoscopy. Patients were censored if they were without diagnosed hernia recurrence at the last date of data extraction from the EMR. To assess differences in time to recurrence, we performed time-to-event analysis using the Kaplan–Meier estimator with the log rank test. In addition, we performed a sensitivity analysis using inverse probability of treatment weighting (IPTW) to reduce confounding between treatment groups and improve covariate balance. IPTW-adjusted analyses were compared with the unadjusted analyses to assess whether weighting materially altered the observed associations. Because the IPTW-adjusted and unadjusted results were directionally similar and did not change the study conclusions, unadjusted results are presented in the main text.

## 3. Results

### 3.1. Total Cohort

Overall, 413 patients [73% female, median age 66 (58–73) years] underwent laparoscopic repair of large PEH over the study entry period (Table 1). The median number of sutures used, which was considered as a surrogate of defect size, was four (IQR: 3–4). Fifty-one percent (n = 212/413) had partial and 49% (n = 201/413) had complete fundoplication. Overlay of porcine SIS mesh was used in 78% (n = 323/413) of the cases, and 51% (n = 209/413) had a PEG placed for gastropexy. The mean follow-up time of the entire cohort was 5.02 ± 4.94 years (median 4.5 years, IQR 2.8–7.1 years).

In total, 69% (n = 286/413) of patients had postoperative radiographic and/or endoscopic studies, while 31% (n = 127/413) did not undergo objective postoperative imaging or endoscopic surveillance. In the primary analysis, patients without postoperative imaging or endoscopy were classified as having no objective recurrence. A recurrence was noted in 19.9% (n = 82/413) of patients, while 1% (n = 5/413) underwent reoperation.

### 3.2. Fundoplication Type

The mean follow up was 3.36 +/− 3.35 and 6.78 +/− 5.68 years for the partial and complete fundoplication subgroups, respectively (median 3.0 years, IQR 1.8–5.5; median 6.2 years, IQR 3.5–9.0, respectively). Patients having partial fundoplication were older than those having complete fundoplication [median 70 (IQR: 63–76) years vs. 63 (IQR: 56–69) years, *p* < 0.0001] and had a larger number of hiatal sutures used [median 4 (IQR: 3–5) sutures vs. 3 (IQR: 3–4) sutures, *p* = 0.01] (Table 2). There were similar rates of 30-day overall complications (*p* = 0.56) and Clavien–Dindo grade III-V complications (*p* = 0.09), but a smaller percentage of patients in the partial fundoplication group had long-term postoperative studies done (63.2% vs. 75.6%, *p* = 0.008) (Table 2).

**Figure 1:**
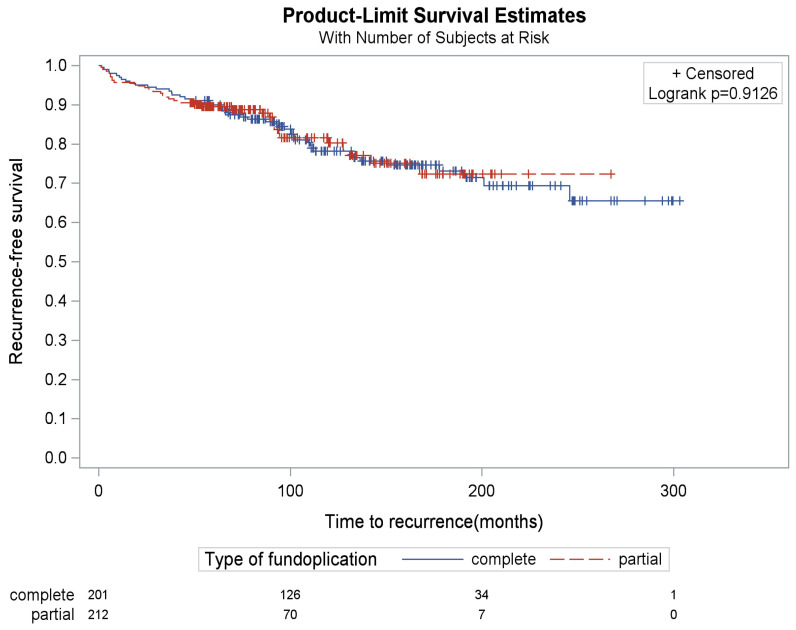
Kaplan–Meier curves of recurrence by repair type (partial vs. complete).

A radiographic or endoscopic recurrence was noted in 16.5% (n = 35/212) and 22.9% (n = 46/201) of the partial and complete fundoplication subgroups, respectively (Table 3), but with similar time to recurrence (*p* = 0.91) (Figure 1). Comparison of these percentages did not identify significant differences in radiographic or endoscopic recurrence rates between the two subgroups (*p* = 0.09). Of the subset of patients who had postoperative radiographic or endoscopic testing, the recurrence rate was similar (*p* = 0.51) (Table 3). Recurrence risk was 16.5% and 23.4% in patients with complete versus partial fundoplication, respectively, with the risk difference being 6.9% (95% CI: −0.8%, 14.6%, *p* = 0.08)

There were five reoperations for symptomatic hernia recurrence, all of which occurred in the complete fundoplication group, and comparison of these numbers demonstrated that patients with complete fundoplication were statistically more likely to undergo reoperation compared to those undergoing partial fundoplication (*p* = 0.03) (Table 3). Unfortunately, due to medical record transitions over the time of the series, the specific symptoms associated with these reoperations were not consistently recorded.

### 3.3. Use of Mesh Reinforcement

The mean follow up was 4.09 +/− 3.60 and 8.36 +/− 7.18 years for the mesh and non-mesh subgroups, respectively (median 3.8 years, IQR 2.1–6.2, and median 7.5 years, IQR 4.0–11.0, respectively). Patients who received overlay absorbable mesh were of similar age to those who had no mesh [median 67 (IQR: 59–74) years vs. 63 (IQR: 56–72) years, *p* = 0.11] and had a larger number of hiatal sutures used [median 4 (IQR: 3–5) sutures vs. 3 (IQR: 2–4) sutures, *p* < 0.0001] (Table 4). There were similar 30-day overall complications (*p* = 0.40) and Clavien–Dindo grade III-V complications (*p* = 0.35), and a similar percentage having long-term postoperative studies done in the mesh and non-mesh groups (*p* = 0.52) (Table 4).

**Figure 2:**
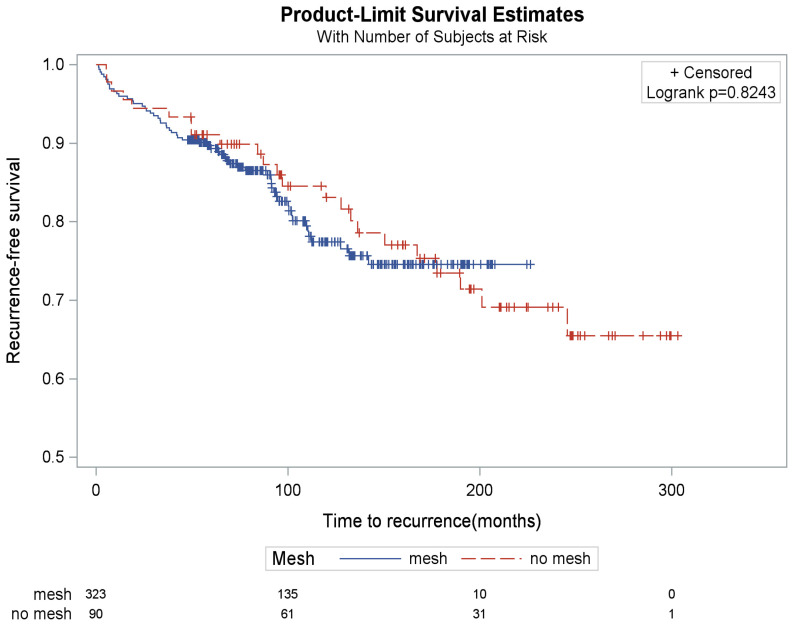
Kaplan–Meier curves of recurrence by mesh implementation (mesh vs. non-mesh).

A radiographic or endoscopic recurrence was noted in 17.6% (n = 57/323) and 24.4% (n = 22/90) of the mesh and non-mesh subgroups, respectively. Comparison of these percentages did not identify significant differences in recurrence rates between the two subgroups (*p* = 0.14) (Table 5). There was a similar time to recurrence between groups (*p* = 0.82) (Figure 2). When focusing within the subset of patients who had postoperative radiographic or endoscopic testing, the recurrence rate was similar (*p* = 0.28) (Table 5). Recurrence risk was 18.3% and 25.6% in patients with mesh versus non-mesh, respectively, with the risk difference being 7.3% (95% CI: −2.7%, 17.2%, *p* = 0.151)

Three reoperations were noted in the mesh subgroup and two in the non-mesh subgroup, but no significant difference was identified (*p* = 0.30) (Table 5).

### 3.4. Use of PEG

The mean follow up was 3.63 +/− 3.28 and 6.45 +/− 5.87 years for the PEG and no-PEG subgroups, respectively (median 3.2 years, IQR 1.9–5.8, and median 6.0 years, IQR 3.2–9.5, respectively). Patients who received a PEG were older than those who did not receive a PEG [median 69 (IQR: 62–76) years vs. 64 (IQR: 56–71) years, *p* < 0.0001] and had a larger number of hiatal sutures used [median 4 (IQR: 3–5) sutures vs. 3 (IQR: 3–4) sutures, *p* = 0.0001] (Table 6). There were more 30-day complications in the PEG group (14.8% vs. 7.8%, *p* = 0.03), although the Clavien–Dindo grade III-V complications were similar (*p* = 0.12) between groups. There were eight complications related to the tube or PEG site (two dislodgements requiring reoperation, three buried bumpers requiring radiologic or endoscopic repositioning, and three superficial site infections). A similar percentage of patients in the PEG and no-PEG groups had long-term postoperative studies done (65.1% vs. 73.5%, *p* = 0.07) (Table 6).

**Figure 3:**
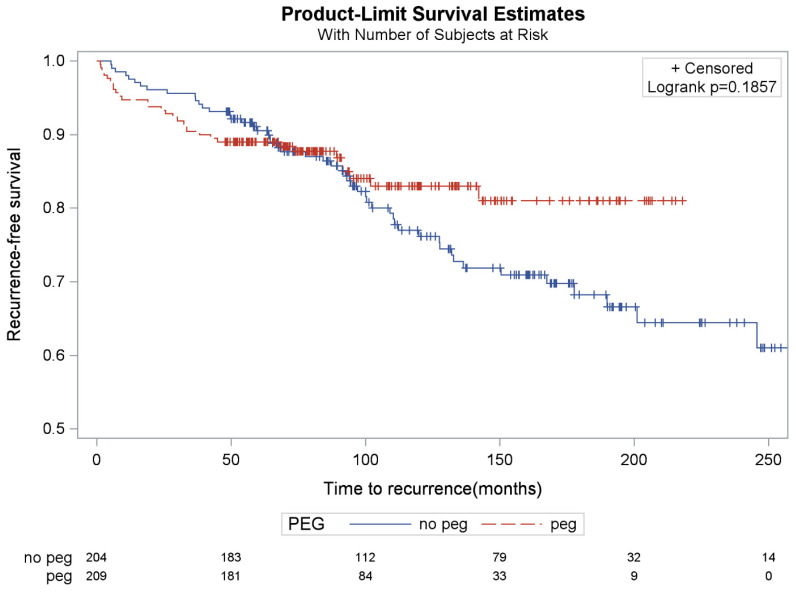
Kaplan–Meier curves of recurrence by PEG tube implementation (PEG vs. No PEG).

A radiographic or endoscopic recurrence was noted in 14.8% (n = 31/209) and 23.5% (n = 48/204) of the PEG and no-PEG subgroups, respectively. Comparison of these percentages demonstrated that the PEG subgroup had significantly fewer recurrences compared to the no-PEG subgroup (*p* = 0.013) (Table 7). Time to recurrence was not statistically different between groups (*p* = 0.19) (Figure 3). Among the subset of patients who underwent postoperative radiographic or endoscopic testing, the lower recurrence rate in the PEG group persisted (22.8% vs. 32.0%), although the difference was attenuated and reached only borderline statistical significance (*p* = 0.0502) (Table 7). Because postoperative testing was more likely to be obtained in patients with symptoms or clinical concern for recurrence, this subset may overrepresent higher-risk patients. Recurrence risk was 14.8% and 25.0% in patients with PEG versus non-PEG, respectively, with the risk difference being 10.2% (95% CI: 2.5%, 17.8%, *p* = 0.092)

One reoperation was noted in the PEG subgroup (a para-hiatal hernia) and four were noted in the no-PEG subgroup (all hiatal recurrences), but no significant differences were identified (*p* = 0.21) (Table 7).

To provide a concise overview of outcomes across all adjunctive strategies, Table 8 summarizes recurrence and reoperation rates for fundoplication type, mesh implementation, and PEG placement. This summary enables rapid comparison of the impact of each intervention on long-term outcomes.

## 4. Discussion

Compared to prior large retrospective case series [18] and randomized control trials [19], our study presents, to the best of our knowledge, one of the largest, long-term, single-institution experiences with laparoscopic repair of large PEHs. Over an average follow-up period exceeding 5 years, 19.9% of the overall cohort were classified as having radiographic or endoscopic recurrence in the primary analysis, although this may underestimate the true recurrence rate because postoperative imaging or endoscopy was not uniformly obtained [20]. These results demonstrate the durability of the laparoscopic approach, even in an older patient population with large paraesophageal hernias.

Of the 413 patients included, 73% were female and the mean age was 65 years. SIS overlay mesh was used in most cases (78% mesh vs. 22% no mesh), while the distribution of partial and complete fundoplications (51% partial vs. 49% complete) and the utilization of gastropexy by PEG (51% PEG vs. 49% no-PEG) were more evenly distributed. We found that neither type of fundoplication nor use of absorbable mesh were significantly associated with PEH recurrence on imaging or endoscopy. However, our cohort of patients with concomitant PEG placement had significantly lower rates of recurrence compared to those that did not have a PEG placed. Interestingly, comparison of the reoperation rates indicated that patients undergoing a complete fundoplication were more likely to have a reoperation, while mesh and PEG placement did not seem to significantly impact reoperation rates. Although not randomized, this large case series and the systematic application of these adjunctive techniques for repair of large paraesophageal hernias allows additional discussion regarding their safety and potential for preventing recurrence and reoperation.

### 4.1. Fundoplication Type

Endoscopic or radiographic recurrence rates did not differ significantly between patients undergoing partial versus complete fundoplication. Taken together, our results and those of others [19,21,22,23,24], indicate that type of fundoplication may not impact hernia recurrence rates. This suggests that differences in other areas, such as preoperative symptoms or disease burden, known or suspected esophageal dysmotility, postoperative medication requirements, or long-term QoL rather than hernia recurrence rates, should drive decisions regarding the type of fundoplication. Unfortunately, the current literature comparing symptom response and QoL outcomes of patients undergoing partial versus complete fundoplication is heterogeneous and at times conflicting, likely due in part to the utilization of different questionnaires and scales when assessing postoperative symptoms [19,23]. This inconsistency limits the ability to collate cumulative data for meta-analysis Additionally, differences in functional outcomes after partial versus complete fundoplication have been identified [19]; however, these findings have not been duplicated in other evaluations [21,23]. Universal adoption of well-validated questionnaires and participation in clinical outcome collaboratives to pool operative data and clinical outcomes could improve comparisons and better guide the choice of fundoplication to optimize patient outcomes.

Although recurrence rates did not vary by fundoplication type, we found significantly more reoperations occurred in the complete fundoplication group, even though these patients were younger, with smaller hernias (based on number of sutures utilized to close the crura). This finding raises the question of whether reoperations occur because a complete fundoplication has fewer points of fixation to the esophagus. In fact, a recent meta-analysis of randomized controlled trials indicated that partial posterior fundoplication may be more durable and cause less dysphagia compared to complete fundoplication [25]. We postulate that reduced slippage and less chance for intra-wrap herniation, as well as less dysphagia, are likely drivers of the lower reoperation rate noted in the partial fundoplication group.

### 4.2. Use of Mesh Reinforcement

In our series, use of SIS mesh overlay was applied in patients with larger hernias; however, neither recurrence nor reoperation rate varied based on its use. Several previous multi-center [24] and single-center comparative studies [26,27]; single-center and single-arm studies [28,29,30,31]; registry analyses [32] and meta-analyses [33] have supported the safety and benefit of absorbable mesh implementation in PEH repair. Our results align with published studies supporting that mesh implementation does not impact long-term hiatal hernia recurrence, but may have value if there are concerns for short-term recurrence [30,34,35,36,37]. Although absorbable mesh was frequently used in our series, we did not observe a long-term reduction in recurrence. Prior studies have suggested potential short-term benefits, but these were not sustained over time. From a practical standpoint, mesh adds direct material costs, which at our institution historically ranged from approximately $800–$1200 per case, not including potential costs related to complications. Given the lack of demonstrated long-term benefit in our cohort, routine use of absorbable mesh may not be cost-effective, and selective or limited implementation should be considered based on individual patient risk factors. The lack of benefit in terms of long-term recurrence, in addition to cost considerations, suggest other adjunctive techniques are needed to reduce the long-term risk of recurrence of large paraesophageal hernias [38]. For example, use of a diaphragm-relaxing incision when indicated [39], and utilization of pledgets to reinforce the hiatal sutures have both shown potential benefits in reducing paraesophageal hernia recurrence [40].

### 4.3. Use of PEG

Finally, our results suggest that patients who had a PEG for gastropexy at the time of laparoscopic repair of large PEHs had a lower endoscopic and radiographic recurrence rate than patients where no PEG was placed, despite patients being older and having larger paraesophageal hernias in the PEG cohort. This difference was significant in our overall cohort analysis, and at the borderline of significance in the subset analysis focusing only on patients that had endoscopic or radiographic studies performed. These findings are in concordance with previously published results supporting placement of PEGs as feasible and safe [41], and leading to a lower risk of paraesophageal hernia recurrence [42], and more recent studies similarly supporting the use of a suture gastropexy to reduce the risk of paraesophageal hernia recurrence [43]. That said, due to low numbers, we did not see a difference in reoperation rates for PEG patients in our study.

Complications were more likely in PEG compared to no-PEG patients. While there were some PEG-related complications, it is likely that differences in age and hernia complexity in this non-randomized study contributed substantially to this difference, as well. Understanding this risk of PEG-related complications, such as dislodgement or surgical site infection, surgeons could consider selective utilization of PEG gastropexy for large paraesophageal hernias. PEG gastropexy may limit the potential for paraesophageal hernia recurrence while also supporting patients’ postoperative recovery by allowing access for venting or nutritional support, and PEG safety can be improved by the addition of Stamm sutures to limit dislodgements. Clearly, there is need for multi-center, randomized, controlled studies to better understand the benefits of PEG gastropexy in this patient population, given that causality cannot be inferred from a retrospective study.

### 4.4. Limitations

Limitations of the study include the bias inherent to retrospective chart review of a single health system’s EMR, with selection bias inherent to the patients who followed up within the health system and after the date of the rollout of the EMR since prior paper chart data, such as detailed operative data including the specific indications for reoperation, were incompletely captured. In addition, the study may have been underpowered to detect differences between treatment groups where no recurrence difference was identified (fundoplication type and mesh usage). An additional limitation is that postoperative radiographic or endoscopic surveillance was not uniformly obtained. Of the 413 patients, 286 (69%) underwent postoperative radiographic and/or endoscopic evaluation, while 127 (31%) did not. In the primary analysis, patients without postoperative imaging or endoscopy were assumed not to have an objective recurrence, which may underestimate the true recurrence rate by preferentially classifying asymptomatic or unevaluated patients as non-recurrent. To address this, we performed a sensitivity analysis limited to patients with postoperative radiographic or endoscopic testing (Table 7). In that restricted analysis, the lower recurrence rate in the PEG group persisted (22.8% vs. 32.0%), although the difference was attenuated and became borderline statistically significant (*p* = 0.0502). Importantly, patients who underwent postoperative imaging or endoscopy were likely enriched for symptoms or clinical concern for recurrence, such that restricting analysis to only tested patients may itself bias recurrence estimates upward. Thus, the true effect of incomplete surveillance on the PEG versus no-PEG comparison is uncertain, but the persistence of the directional association in the tested subset suggests that the finding was not solely driven by inclusion of patients without objective follow-up.

Over the long course of this single-institution series, the medical records evolved from paper charting through multiple EMRs; therefore, potential data loss in these transitions cannot be excluded. Lastly, over the course of the series, PEG gastropexy was increasingly utilized for patients with very large PEHs as the surgeon came to recognize the benefits of gastric decompression and likely protection of the repair. Despite these limitations, our results support a potential benefit of PEG placement to anchor the stomach and give support for further exploration of this option in a prospective manner.

## 5. Conclusions

Our study constitutes one of the largest single-center case series of laparoscopic large PEH repairs performed by a single surgeon. In this series, adjunctive measures such as use of absorbable mesh at the hiatus and use of a PEG gastropexy were utilized selectively for those hernias felt to be at higher risk of recurrence. Fundoplication type and use of absorbable mesh did not have a significant impact on rates of radiographic or endoscopic recurrence, although partial fundoplication was associated with fewer reoperations. Interestingly, a PEG gastropexy at the time of laparoscopic large PEH repair was associated with fewer radiographic or endoscopic recurrences at a mean follow-up of greater than 12 years. While there were statistically more complications in the PEG group, the rate of severe complications was low and similar to the no-PEG group. Further studies are needed to explore the impact of fundoplication type, mesh overlay and PEG gastropexy before firm conclusions can be established.

## Figures and Tables

**Table 1:** Baseline characteristics of the entire cohort.

	Total Cohort (N = 413)
Characteristic	
Median Age Years (IQR)	66 (58–73)
Sex	
Female N (%)	302 (73)
Male N (%)	111 (27)
Median Hiatal Sutures Used N (IQR)	4 (3–4)
Mean Overall Follow-Up Months (SD)	152.9 (150.3)
Type of Fundoplication	
Partial Fundoplication N (%)	212 (51)
Complete Fundoplication N (%)	201 (49)
Mesh Placement	
Mesh N (%)	323 (78)
No Mesh N (%)	90 (22)
PEG Tube Placement	
PEG N (%)	209 (51)
No-PEG N (%)	204 (49)

IQR: Interquartile range. SD: Standard deviation.

**Table 2:** Comparison of cohorts based on fundoplication type.

	Partial Fundoplication (N = 212)	Complete Fundoplication (N = 201)	*p*-Value
Characteristic			
Median age years (IQR)	70 (63–76)	63 (56–69)	<0.01 *
Median hiatal sutures N (IQR)	4 (3–5)	3 (3–4)	0.01 *
Sex			
Female N (%)	156 (51.7)	146 (48.3)	0.91
Male N (%)	56 (50.5)	55 (49.6)	
Complications			
No complication N (%)	186 (87.7)	180 (89.6)	0.56
Any complication N (%)	26 (12.3)	21 (10.4)	
Clavien–Dindo grade ≥ III N (%)	11 (5.2)	4 (2.0)	0.09
^1^ Postop test done N (%)	134 (63.2)	152 (75.6)	<0.01 *

IQR: Interquartile range. ^1^ Number of patients with a documented postoperative barium swallow or upper gastrointestinal tract endoscopy. * Indicates statistical significance.

**Table 3:** Recurrence and reoperation rates in patients with partial versus complete fundoplication.

	**Partial Fundoplication (N = 212)**	**Complete Fundoplication (N = 201)**	***p*-Value**
Recurrence in all patients N (%)	35 (16.5)	46 (22.9)	0.09
Reoperations N	0	5	0.03 *
	**Partial Fundoplication (N = 134)**	**Complete Fundoplication (N = 152)**	***p*-Value**
^1^ Recurrence in patients tested N (%)	35 (26.1)	46 (30.3)	0.51

^1^ Recurrence noted on postoperative barium swallow or upper gastrointestinal tract endoscopy. Of note, detailed indications for reoperation were not consistently documented due to EMR transitions. * Indicates statistical significance.

**Table 4:** Comparison of cohorts based on mesh implementation.

	Mesh (N = 323)	No Mesh (N = 90)	*p*-Value
Characteristic			
Median age years (IQR)	67 (59–74)	63 (56–72)	0.11
Median hiatal sutures N (IQR)	4 (3–5)	3 (2–4)	<0.01 *
Sex			
Female N (%)	246 (81.5)	56 (18.5)	0.01 *
Male N (%)	77 (69.4)	34 (30.6)	
Complications			
No complication N (%)	284 (87.9)	82 (91.1)	0.40
Any complication N (%)	39 (12.1)	8 (8.9)	
Clavien–Dindo grade ≥ III N (%)	14 (4.3)	2 (2.2)	0.35
^1^ Postop test done N (%)	221 (68.4)	65 (72.2)	0.52

IQR: Interquartile range. ^1^ Number of patients with a documented postoperative barium swallow or upper gastrointestinal tract endoscopy. * Indicates statistical significance.

**Table 5:** Recurrence and reoperation rates in patients with and without mesh implementation.

	**Mesh** **(N = 323)**	**No Mesh** **(N = 90)**	***p*-Value**
Recurrence in all patients N (%)	57 (17.6)	22 (24.4)	0.14
Reoperations (N)	3	2	0.30
	**Mesh** **(N = 221)**	**No Mesh** **(N = 65)**	***p*-Value**
^1^ Recurrence in patients tested N (%)	57 (25.8)	22 (33.8)	0.28

^1^ Recurrence noted on postoperative barium swallow or upper gastrointestinal tract endoscopy. Of note, detailed indications for reoperation were not consistently documented due to EMR transitions.

**Table 6:** Comparison of cohorts based on PEG implementation.

	PEG (N = 209)	No PEG (N = 204)	*p*-Value
Characteristic			
Median age years N (IQR)	69 (62–76)	64 (56–71)	<0.01 *
Median hiatal sutures N (IQR)	4 (3–5)	3 (3–4)	<0.01 *
Sex			
Female N (%)	149 (49.3)	153 (50.7)	0.44
Male N (%)	60 (54.1)	51 (46.0)	
Complications			
No complication N (%)	178 (85.2)	188 (92.2)	0.03 *
Any complication N (%)	31 (14.8)	16 (7.8)	
Clavien–Dindo grade ≥ III N (%)	11 (5.3)	5 (2.5)	0.12
^1^ Postop test done N (%)	136 (65.1)	150 (73.5)	0.07

IQR: Interquartile range. ^1^ Number of patients with a documented postoperative barium swallow or upper gastrointestinal tract endoscopy. * Indicates statistical significance.

**Table 7:** Recurrence and reoperation rates in patients with and without PEG placement.

	**PEG** **(N = 209)**	**No PEG** **(N = 204)**	***p*-Value**
Recurrence in all patients N (%)	31 (14.8)	48 (23.5)	0.01 *
Reoperations N	1	4	0.21
	**PEG** **(N = 136)**	**No PEG** **(N = 150)**	***p*-Value**
^1^ Recurrence in patients tested N (%)	31 (22.8)	48 (32.0)	0.05 *

^1^ Recurrence noted on postoperative barium swallow or upper gastrointestinal tract endoscopy. * Indicates statistical significance. Of note, detailed indications for reoperation were not consistently documented due to EMR transitions.

**Table 8:** Summary of recurrence and reoperation rates by adjunctive strategy.

Adjunctive Strategy	Recurrence (%)	Reoperation (%)	Key Observation
Fundoplication Type	Partial: 16.5% Complete: 22.9%	Partial: 0% Complete: 2.5%	Reops higher with complete fundoplication
Mesh Overlay	Mesh: 17.6% No Mesh: 24.4%	Mesh: 0.9% No Mesh: 2.2%	Mesh used for larger hernias; long-term similar
PEG Gastropexy	PEG: 14.8% No PEG: 23.5%	PEG: 0.5% No PEG: 2%	Lower recurrence with PEG; minor short-term risk

Recurrence = radiographic or endoscopic evidence ≥30 days post-op. Reoperations = symptomatic recurrence requiring surgery.

## Data Availability

The data presented in this study are available on request from the corresponding author due to privacy.

## Abbreviations

PEH	Paraesophageal Hernia
PEG	Percutaneous Endoscopic Gastrostomy
GERD	Gastroesophageal Reflux Disease
HH	Hiatal Hernia
LES	Lower Esophageal Sphincter
QoL	Quality of Life
SAGES	Society of American Gastrointestinal and Endoscopic Surgeons
SSI	Superficial Skin Infections
PE	Pulmonary Embolism
SIS	Small Intestinal Submucosa
